# A population study on the time trend of cigarette smoking, cessation, and exposure to secondhand smoking from 2001 to 2013 in Taiwan

**DOI:** 10.1186/s12963-016-0109-x

**Published:** 2016-11-05

**Authors:** Chi-Yung Chiang, Hsing-Yi Chang

**Affiliations:** 1 Institute of Population Health Sciences, National Health Research Institutes, 35 Keyan Road, A3223, Zhunan Town, Maoli County 350 Taiwan, Republic of China; 2 Institute of Public Health, National Yang-Ming University, 35 Keyan Road, A3223, Zhunan Town, Maoli County 350 Taiwan, Republic of China

**Keywords:** Smoking prevalence, Secondhand smoking, Smoking cessation, Time trend, NHIS, Taiwan

## Abstract

**Background:**

In 2001, the National Health Interview Survey (NHIS) commenced in Taiwan. This survey, conducted on a sample of the whole Taiwanese population, is nationally representative and has a high response rate (>80 %). As a result, the four already completed surveys from 2001 to 2013 can be used to investigate the time trend of smoking prevalence, the rate of cessation, and exposure to secondhand smoking.

**Methods:**

There were 72918 adults combined from the 2001, 2005, 2009 and 2013 National Health Interview Surveys (NHIS). Smoking status, exposure to secondhand smoking, and smoking cessation were asked, as well as demographic characteristics and other variables. Statistical analyses with sampling weights were carried out using SAS and SUDAAN.

**Results:**

In males, the prevalence of smoking significantly decreased (rates in 4 surveys were 44.4 %, 44.6 %, 38.9 %, and 34.2 %, respectively). Since 2005 the rate of smoking cessation increased significantly (*p* = 0.033). The odd ratio (OR) exposure of secondhand among non-smokes (OR) in 2009 and 2013 were 0.96 (CI = 0.85–1.08) and 0.78 (CI = 0.70–0.88) comparing to 2005. In females, the prevalence of smoking was stable over time. The rate of smoking cessation only appeared significantly high in the older age group. The OR for exposure to secondhand smoking were 0.81 (CI = 0.74–0.89) and 0.68 (CI = 0.62–0.74), for 2009 and 2013 comparing to 2005, respectively.

**Conclusion:**

Early anti-smoking legislation in Taiwan might have raised the awareness of the harm of smoking. However, the implementation of the Tobacco Hazards Prevention Act (THPA) in 2009 had great contribution to the reduction of smoking rate, especially in males.

**Electronic supplementary material:**

The online version of this article (doi:10.1186/s12963-016-0109-x) contains supplementary material, which is available to authorized users.

## Background

Research shows that as well as damaging the respiratory system [1], smoking is strongly associated with cancer and cardiovascular diseases [2], and even causes harm to the urinary and reproductive systems. [3] According to WHO statistics, worldwide on average one person dies from smoking every six seconds and those who use tobacco products lose on average 15 years of life expectancy [4]. In addition, those who consume large quantities of tobacco or start smoking at an earlier age are more likely to have difficulty quitting [5].

To combat the problem of smoking related harm globally, in 2005 the WHO passed the Framework Convention on Tobacco Control (FCTC). This framework aims to decrease the risk to human health from the consumption of tobacco products through international standards in tobacco pricing and taxes, tobacco advertising and sponsorship, and tobacco product labeling, reducing illegal trade of tobacco products, and decreasing exposure to secondhand smoke. The message has spread all over the world. Taiwan is one of the countries with a national tobacco control campaign.

Anti-smoking act in Taiwan began in 1986 with the enactment of an executive order prohibiting smoking in public places. Under this order, offenders could be fined NTD 3000 (~100USD) under the Maintenance of Public Order Act. This action had a short term effect on smoking rates but they had returned to previous levels within 2 years [6] . It was possibly due to a lack of appropriate human resources to enforce it and a lack of accompanying strategies, this order was not effectively complied with. The first legislation of THPA was passed in 1997. This old version of THPA only indicate what should be done. It did not state clearly about the punishment. Nor did it allocate enough manpower and funds. Thus the enforcement was low. A NTD 5 (~0.16USD) surcharge was added to each cigarette packet only from 2002 (and increased to NT$10 in 2006). Manpower and funds were made available for promotion of outpatient smoking cessation clinics in 2002, followed by a quit line (2003), smoke-free schools (2003), smoke-free workplaces (2003), and smoke-free restaurants (2004). The latest THPA was passed on June 15, 2007. The president announced the amendment on July 11, 2007. There was an 18-month preparation before the actual execution of THPA on January 11, 2009. During the preparation period, public education and promotion were conducted. The information was conveyed to the public via many media, i.e. TV advertisements, poster in public places, etc.

As in many Asian countries, smoking in Taiwan has traditionally been a behavior undertaken mostly by males. By the turn of the millennium, smoking rates among Taiwanese men approached the levels observed in the United States [7] and the United Kingdom in the mid-1950s [8]. However, the introduction of effective advertising for international cigarette brands,that included a focus on encouraging women to start smoking, was associated with a rise in smoking by young women, similar to what occurred in the United States with the Virginia Slims campaign [9]. The introduction of the first mass media-led tobacco control program in Taiwan in 2007 might be expected to have the result that the 1980s “Quit for Life” tobacco control program had in Australia which was a sudden shift in prevalence associated with smoking [10]. A similar effect occurred in the United States in the 1950s with the dissemination of the health consequences of smoking, wherein the decline in prevalence was associated with increased cessation particularly in middle aged and older smokers [11]. We hypothesize that there would be a sudden increase in cessation among middle aged and older smokers associated with the introduction of Taiwan’s 2007 tobacco control campaign. Also, we hypothesize that the implementation of a ban on cigarette advertising would halt the rise in smoking among young Taiwanese women. Our third hypothesis is that the implementation of the smoke-free policies would reduce levels of exposure to secondhand smoke among nonsmokers especially at work.

In 2001 the National Health Interview Survey (NHIS) commenced in Taiwan. This survey, conducted on a sample of the whole Taiwanese population, is nationally representative and has a high response rate (>80 %). As a result, the four already completed surveys from 2001, 2005, 2009, and 2013 can be used to investigate the time trend of smoking prevalence, the rate of cessation, and exposure to secondhand smoking, furthermore, to confirm the above mentioned hypotheses. This is a good chance to examine the effectiveness of the efforts in combat smoking in Taiwan.

The aim of the present study was to analyse data from the 2001, 2005, 2009, and 2013 surveys to examine changes in rates of smoking, smoking cessation and exposure to secondhand smoke.

## Methods

### Data

The present study analyses data from the 2001, 2005, 2009, and 2013 NHIS. The sample population was the database of Taiwanese registered households the year before survey. The sample was chosen using a multi-stage stratified systematic sampling design, and sampling was carried out in each stratum using Probability Proportional to Size (PPS). In 2001, the whole Taiwan area was divided into seven strata [12]. Then, townships/districts were selected with PPS. Households were the basic sampling unit (all members of the selected households were interviewed). This resulted in an equal probability sample [12]. In the 2005, 2009 and 2013 surveys each city/county was a stratum, individuals were the basic sampling unit. They were unequal probability samples. As a result, these samples were weighted for analysis. Weights was calculated using the Taiwanese sample weights provided by the National Health Interview Survey working group. The response rates were 93.8 %, 85.6 %, 84.0 %, and 75.2 % for 2001, 2005, 2009, and 2013, respectively. The decline of response rates might be due to increasing crime rates and decreasing accessibility of household registry data. The data collection was approved by the Institution Review Board of National Health Research Institutes. Further details of the sampling design, questionnaire content, and survey process can be found elsewhere [13, 14] and on the NHIS website (http://nhis.nhri.org.tw/). The data is open for application on the same website.

### Variables

Smoking status, cessation and exposure to secondhand smoke to those aged 18 years and over. There were 16136 respondents in the 2001 survey, 18529 in the 2005 survey, 19796 in the 2009 survey, and 18457 in the 2013 survey.

Respondents were asked have they ever smoked. If they answered ‘never’ or ‘only few times’, then they were considered non-smokers. Otherwise, they were asked whether they have smoked more than 100 cigarettes, followed by the age of initiation, duration, and current status (Additional file 1). In this study we used the US Centers for Disease Control and Prevention (CDC) standard definition of a smoker as someone who has smoked 100 or more cigarettes [15]. Smoking status was defined as: (1) Non-smoker: including never smokers, and those who have smoked but less than 100 cigarettes; (2) Current Smoker: those who have ever smoked at least 100 cigarettes and are currently still smoking almost every day or on some days; and (3) Ex-smoker: those who have smoked 100 or more cigarettes but who have quit smoking. Cessation rate (quit ratio) is defined as ex-smokers/all those who have ever smoked 100 or more cigarettes [16, 17].

In terms of exposure to secondhand smoke, this was not assessed in the 2001 survey. Starting in 2005, we first asked whether they have been exposed to secondhand smoke, then asked the places for exposure. In the other three surveys exposure to secondhand smoke in the home environment, at friends and relatives’ homes, school and the workplace, other indoor public places, and outdoor places were asked in 2009 and 2013 (Additional file 1). Secondhand smoke to non-smokers is of concern [18, 19]. Thus, the analysis of secondhand smoke was limited to current non-smokers. The 2005 survey also included items about exposure in restaurants and other public places. The reason for asking restaurant was that Taiwanese government wanted to see the effectiveness of the policy on smoking-free restaurant. Besides that, the 2005 question asked the public areas as a whole (Additional file 1). To ensure homogeneity of comparisons, we carried out comparisons between the locations of home, friend and relatives’ homes, schools, and workplaces.

The actual date of birth was asked to the respondents. Age calculated to the first day of the interview and was categorized into the following four groups: 18–24, 25–39, 40–64, and 65 years and over. Education level refers to the highest level of education obtained by the respondent and was grouped as below senior high school (includes elementary school and below, junior high school, senior high school or vocational school (1 − 3 years) and university and above (includes university, 4- and 5-year junior colleges, 2- and 3-year junior colleges, 2 and 4-year technical institutes, open universities, and graduate schools). Household mean monthly income refers to the mean monthly income in the past year received by the household and was grouped as ≦NT$29,999 (<1000 USD), NT$30,000–99,999 (1000 ~ 3333 USD), and ≥ NT$100,000 (>3333 USD). Marital status was grouped as never married, married and living with spouse, and other (including married but not living with spouse, divorced, widowed, formally separated, and living with a partner).

Urbanization levels were based on the results of Liu et al’s work to group Taiwanese townships/districts into homogenous groups based on demographic and economic characteristics of the township or districts [20]. In the three surveys the urbanization level was based on the standard 2005 urbanization level of townships. Residential locations were divided into low level of urbanization (includes general townships, townships with a high proportion of older persons, agricultural townships and remote villages), medium level of urbanization (includes medium level urbanization and newly developing townships) and high level of urbanization [21].

### Statistical analyses

Due to the complex sampling design of the NHIS, we used SUrvey DAta ANalysis (SUDAAN) version 11.0 (SAS-Callable) to account for sampling weights and sampling scheme for all statistical testing and for the weighting of the sample to make it nationally representative. We used SAS 9.3 and SUDAAN 11.0 statistical software for statistical analyses. The chi squared test was used to compare smoking and cessation rates between different age groups, secondhand smoke exposure between different locations, and different quantities of cigarettes consumed. Logistic regression analysis was used to examine factors associated with smoking and secondhand smoke exposure. We tested the stability of our estimates and did not present data which was considered unstable. Our criteria for instability were: a) relative standard error (RSE (p ˆ ) > 30 %; or b) RSE(1-p ˆ) > 30 %; or c) n < 50 were labeled [22].

## Results

Characteristics of participants who completed the four surveys are shown in Table 1. Comparison between surveys shows that level of education and household monthly income gradually increased over the three surveys, especially in women (*p* < 0.0001). Individual monthly income and workforce participation also gradually increased over time in women (*p* < 0.0001) (Table 1).

**Table 1:** Characteristics of survey participants

Sex	Male				Female			
Year of survey	2001	2005	2009	2013	2001	2005	2009	2013
Total participants	*N* = 7980	*N* = 9522	*N* = 10093	*N* = 9354	*N* = 8156	*N* = 9349	*N* = 10108	*N* = 9497
Age								
18-24	16.2 %	14.9 %	12.9 %	12.4 %	15.5 %	14.5 %	12.2 %	11.1 %
25-39	32.3 %	32.8 %	31.8 %	29.9 %	32.2 %	32.3 %	31.4 %	29.4 %
40-64	38.2 %	40.1 %	42.6 %	44.7 %	40.0 %	40.8 %	42.9 %	45.0 %
> =65	13.2 %	12.3 %	12.7 %	13.0 %	12.4 %	12.4 %	13.5 %	14.4 %
Level of urbanization								
High	20.6 %	23.2 %	23.2 %	22.9 %	21.3 %	24.6 %	24.2 %	23.7 %
Medium	50.8 %	50.0 %	51.4 %	52.6 %	51.4 %	50.8 %	52.5 %	53.4 %
Low	28.6 %	26.8 %	25.5 %	24.5 %	27.3 %	24.5 %	23.3 %	22.9 %
Education level								
Under high school	70.6 %	65.0 %	60.8 %	55.9 %	76.3 %	69.2 %	64.1 %	59.7 %
college degree or above	29.4 %	35.0 %	39.2 %	44.1 %	23.7 %	30.8 %	35.9 %	40.3 %
Mean monthly household income								
< 30,000	19.6 %	21.5 %	20.9 %	17.9 %	20.2 %	23.8 %	24.4 %	19.7 %
30,000-99,999	62.7 %	61.1 %	61.2 %	60.7 %	62.5 %	59.2 %	57.8 %	59.7 %
≧10,000	17.6 %	17.4 %	17.9 %	21.5 %	17.3 %	16.9 %	17.8 %	20.6 %
Marital status								
Married and living together	61.0 %	58.8 %	58.1 %	58.0 %	60.8 %	58.6 %	55.4 %	55.0 %
Other	8.9 %	9.1 %	9.2 %	9.7 %	16.9 %	16.1 %	18.5 %	19.3 %
Never married	30.1 %	32.1 %	32.7 %	32.3 %	22.3 %	25.3 %	26.1 %	25.7 %
Employment status								
Not currently employed	33.3 %	28.4 %	29.7 %	27.4 %	50.6 %	43.7 %	41.3 %	40.4 %
Employed	66.7 %	71.6 %	70.3 %	72.6 %	49.4 %	56.3 %	58.7 %	59.6 %

Note 1: The value of N refers to the weighted value for the whole of Taiwan

Note 2: Level of urbanization: ‘Medium’ includes newly developing towns, ‘Low’ includes general townships and villages, townships with an ageing population, agricultural towns and remote villages

Note 3: Education level: Elementary school and below includes both literate and illiterate participants. University and technical college includes open universities and open professional colleges

Note 4: Marital status: ‘Other’ includes married but not living together, divorced, widowed, living with a partner, and formally separated

Note 5: Smoking status: ‘Never smoker’ refers to those that have never smoked or have smoked 100 cigarettes or fewer. ‘Smoker’ refers to those who have smoked more than 100 cigarettes and are still currently smoking. ‘Ex- smoker’ refers to those who have smoked more than 100 cigarettes but are no longer currently smoking

### Smoking

**Fig. 1:**
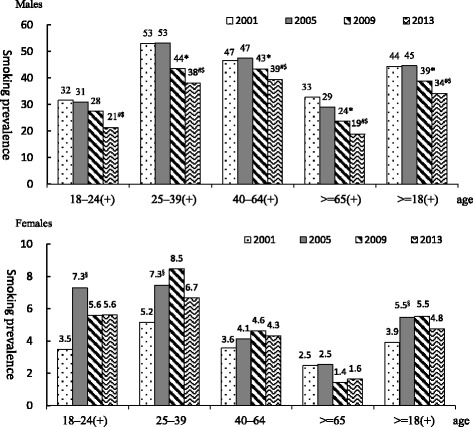
Time trend of prevalence of smoking between 2001 and 2013 by gender and age groups. Note 1: Smoking was defined as having smoked 100 cigarettes or more and having smoked every day or occasionally during the past month. Note 2: Comparisons between surveys were adjusted using SUDAAN. Note 3: (+) indicates a statistically significant difference in the prevalence of smoking in the particular age group between the 4 surveys, § indicates a statistically significant difference between. 2001 and 2005, * indicates a statistically significant difference between 2005 and 2009, #indicates a statistically significant difference between 2009 and 2013, $indicates a statistically significant difference between 2005 and 2013

The prevalence of smoking in Taiwanese men aged 18 years and over was 44.4 % in 2001 and 44.6 % in 2005 (Fig. 1), decreased to 38.9 % in 2009 (*p* < 0.001). It further decreased to 34.2 % in 2013 (*p* < 0.001). This trend was roughly similar in each age group apart from those aged 65 years and over, where the prevalence of smoking decreased over each of the four survey periods (*p* = 0.001) (Fig. 1).

Logistic regression analysis showed that factors associated with smoking (Table 2) included age, education level, level of urbanization, marital status, income, employment status, betel nut chewing and alcohol consumption. After controlling for confounders, the probability of smoking in men in 2009 was significantly lower than that in 2005 odds ratio (OR) = 0.82 (95 % CI:0.75–0.91), it was even lower in 2013 OR = 0.71, (95 % CI: 0.64–0.79). The probability of smoking in women in 2005 was significantly higher than that in 2001, and the prevalence of smoking in women in 2009 was even higher than that in 2005 (OR = 1.26, CI = 1.04–1.51).

**Table 2:** Factors associated with smoking, cessation, and exposure to secondhand smoke

	Smoking			Cessation			Exposure to secondhand smoke		
	*P* value	OR	95 % CI	*P* value	OR	95 % CI	*P* value	OR	95 % CI
					males				
Age	0.00			0.00			0.00		
18–24vs25–40	0.00	0.64	(0.57–0.72)	0.00	0.55	(0.40–0.76)	0.00	1.58	(1.32–1.88)
40–64vs25–40	0.00	0.72	(0.67–0.79)	0.00	2.08	(1.81–2.39)	0.00	0.51	(0.45–0.59)
> = 65vs25–40	0.00	0.43	(0.38–0.49)	0.00	5.82	(4.77–7.11)	0.00	0.30	(0.24–0.36)
college degree or above VS Under high school	0.00	0.48	(0.45–0.52)	0.00	1.41	(1.24–1.62)	0.00	0.71	(0.63–0.80)
Level of urbanization	0.15			0.00			0.63		
Medium vs high	0.33	1.05	(0.95–1.15)	0.00	0.80	(0.69–0.93)	0.42	0.95	(0.83–1.08)
Low vs high	0.43	0.96	(0.86–1.07)	0.00	0.70	(0.60–0.83)	0.89	0.99	(0.86–1.15)
Marital status	0.00			0.00			0.12		
Other vs married and living together	0.00	1.52	(1.36–1.69)	0.00	0.72	(0.62–0.85)	0.04	1.21	(1.01–1.44)
Never married vs married and living together	0.00	1.22	(1.11–1.34)	0.00	0.55	(0.46–0.67)	0.81	1.02	(0.88–1.18)
Household income(USD)	0.03			0.00			0.01		
<1,000 vs 1,000–3,333	0.04	1.10	(1.00–1.20)	0.00	0.81	(0.71–0.93)	0.00	0.82	(0.72–0.93)
>3,333 vs 1,000–3,333	0.18	0.94	(0.86–1.03)	0.10	1.14	(0.97–1.33)	0.57	0.97	(0.86–.09)
Currently employed vs not employed	0.00	1.28	(1.18–1.39)	0.00	0.67	(0.59–0.77)	0.00	2.05	(1.82–2.31)
Chews betel nut vs doesn’t chew betel nut	0.00	4.53	(4.17–4.92)	0.00	0.76	(0.68–0.86)	0.00	2.14	(1.81–2.54)
Drinks alcohol vs doesn’t drink alcohol	0.00	2.40	(2.25–2.57)	0.00	0.60	(0.54–0.67)	0.00	1.60	(1.41–1.81)
Comparison between surveys	0.00			0.00			0.00		
2001VS2005	0.12	1.08	(0.98–1.18)	0.00	0.75	(0.63–0.88)			
2009VS2005	0.00	0.72	(0.66–0.79)	0.00	2.41	(2.06–2.80)	0.50	0.96	(0.85–1.08)
2013VS2005	0.00	0.63	(0.57–0.69)	0.00	2.56	(2.22–2.95)	0.00	0.78	(0.70–0.88)
					females				
	0.00			0.00			0.00		
	0.13	0.84	(0.66–1.05)	0.29	0.70	(0.36–1.36)	0.00	1.66	(1.45–1.91)
	0.00	0.34	(0.29–0.41)	0.13	1.39	(0.90–2.13)	0.00	0.64	(0.58–0.70)
	0.00	0.12	(0.08–0.16)	0.00	4.96	(2.68–9.18)	0.00	0.31	(0.27–0.36)
	0.00	0.17	(0.14–0.21)	0.00	2.11	(1.33–3.33)	0.00	0.68	(0.63–0.75)
	0.00			0.14			0.02		
	0.04	0.83	(0.69–0.99)	0.44	1.18	(0.77–1.81)	0.09	1.09	(0.99–1.19)
	0.00	0.48	(0.38–0.60)	0.33	0.77	(0.45–1.32)	0.01	1.17	(1.05–1.31)
	0.00			0.01			0.00		
	0.00	3.31	(2.78–3.95)	0.00	0.48	(0.31–0.74)	0.00	0.83	(0.75–0.92)
	0.00	1.99	(1.59–2.50)	0.42	0.82	(0.50–1.35)	0.84	0.99	(0.89–1.10)
	0.01			0.02			0.49		
	0.10	1.14	(0.97–1.34)	0.63	1.11	(0.72–1.70)	0.75	0.99	(0.91–1.07)
	0.01	0.75	(0.60–0.94)	0.00	2.21	(1.29–3.78)	0.24	0.94	(0.85–1.04)
	0.48	1.05	(0.91–1.22)	0.03	0.65	(0.45–0.95)	0.00	1.60	(1.48–1.72)
	0.00	7.84	(5.79–10.9)	0.78	0.92	(0.52–1.64)	0.00	1.99	(1.38–2.85)
	0.00	4.59	(3.93–5.36)	0.00	0.45	(0.30–0.69)	0.00	1.92	(1.67–2.21)
	0.00			0.00			0.00		
	0.00	0.67	(0.55–0.81)	0.85	1.05	(0.63–1.76)			
	0.35	1.09	(0.91–1.30)	0.00	3.14	(2.00–4.94)	0.00	0.81	(0.74–0.89)
	0.70	1.04	(0.85–1.27)	0.01	2.77	(1.67–4.58)	0.00	0.68	(0.62–0.74)

Note 1: Logistic regression analyses were adjusted using SUDAAN

Note 2: OR = odds ratio

Note3: Smoking status: ‘Never smoker’ refers to those that have never smoked or have smoked 100 cigarettes or fewer. ‘Smoker’ refers to those who have smoked more than 100 cigarettes and are still currently smoking. ‘Ex-smoker’ refers to those who have smoked more than 100 cigarettes but are no longer currently smoking

Note4: Secondhand smoking was analyzed among non-smokers who were non-smokers, or ex-smokers

### Smoking cessation

**Fig. 2:**
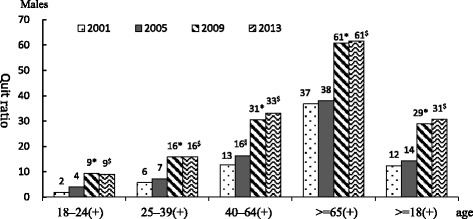
Time trend of quit ratio in men between 2001 and 2013. Note 1: Cessation rate (quit ratio) is defined as ex-smokers/all those who have ever smoked 100 or more cigarettes. Note 2: Comparisons between surveys were adjusted using SUDAAN. Note 3: (+) indicates a statistically significant difference in the prevalence of smoking in the particular age group between the 4 surveys, § indicates a statistically significant difference between. 2001 and 2005, * indicates a statistically significant difference between 2005 and 2009, #indicates a statistically significant difference between 2009 and 2013, $indicates a statistically significant difference between 2005 and 2013

Rates of smoking cessation (quit ratio) are shown in Fig. 2. Due to small number of smokers in females, the estimates were not stable (data not shown). Therefore, we presented the quit ratio for men only. The prevalence of smoking cessation in men was 12.4 % in 2001 and 14.3 % in 2005. However, in 2009 this increased to 28.9 %, then 30.6 % in 2013. In terms of age, smoking cessation rates were higher in men aged 45 − 64 years in 2005 compared to 2001 (*p* = 0.009). It increased in all age groups between 2009 and 2013.

### Exposure to secondhand smoke

**Fig. 3:**
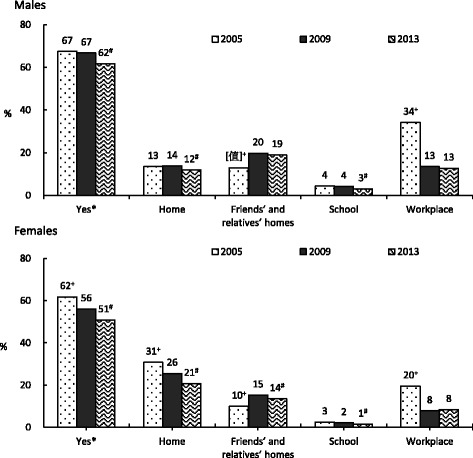
Time trend of exposure to secondhand smoke among non-smokers between 2005 and 2013 by gender and age groups. Note 1: * Were those answering yes to the question ‘has been exposed to secondhand smoke last week’. Note 2: + indicates a statistically significant difference between the rate of exposure to secondhand smoke in 2005 compared to 2009 (*P* < 0.05), # indicates a statistically significant difference between 2009 and 2013 (*P* < 0.05). Note 3: Comparisons between surveys were adjusted using SUDAAN

Locations of exposure to secondhand smoke among non-smokers were compared between the 2005, 2009 and 2013 surveys and are shown in Fig. 3. The exposure to secondhand smoke in the workplace decreased significantly in 2009 and 2013. Logistic regression analysis (Table 2) showed that factors associated with secondhand smoke exposure in both sexes included age, education level, level of urbanization, marital status, mean monthly income, employment status, betel nut chewing, alcohol consumption and smoking behavior. After controlling for these confounders, we found that the likelihood of exposure to secondhand smoke in men was lower in 2013 compared to 2005 (OR = 0.78, 95 % CI = 0.70–0.88). It was not significant in 2009. It decreased from 2005 significantly in females (OR = 0.81, 95 % CI: 0.74–0.89; OR: 0.68 95 % CI: 0.62–0.74 for 2009 and 2013, respectively.

## Discussion

In this study we examined rates of smoking, smoking cessation and exposure to secondhand smoke in Taiwan using data from the 2001, 2005, 2009, and 2013 NHIS. We found little change in smoking prevalence between 2001 and 2005 followed by a dramatic decrease in smoking from 2009 onward. Rates of smoking cessation in men aged 18 years and over were significantly higher in 2005 compared to 2001, especially in men aged 40–64 years. Starting from 2009, rates of smoking cessation in men aged 18 years and over significantly increased across all age groups. The overall rate of exposure to secondhand smoke was lower in 2009 compared to 2005. Possible factors contributing to this decline in smoking prevalence could include government anti-smoking legislation, tobacco pricing, cigarette packaging and various smoking cessation services.

Research by Constantine I [23] found that antismoking legislation needs to be appropriately implemented in order to be effective. Taking the implementation of anti-smoking policy in Greece in 1978–1980 as an example, although smoking prevalence decreased initially, success was short lived as (1) smoking had already become a socially acceptable habit at that time, (2) Greece was producing their own tobacco products, and (3) anti-smoking activities didn’t include public education about smoking harms. After 2 years, the rate of smoking returned to previous levels. A similar phenomenon was observed in Taiwan following the introduction of anti-smoking policy in 1987 [6]. The smoking rate decreased a little in 1987, then rebounded. In 2009, Taiwan implemented an amended THPA. This legislation included the following regulations: (1) Smoke-free areas were extended to include workplaces and the majority of public places, (2) Cigarette packets are required to display one of six possible graphic warning labels, misleading and ambiguous information is banned, and information on constituents must be disclosed. In additions, bans on tobacco advertising were expanded, and (3) Tobacco advertising is comprehensively banned. We found a sharp decrease in smoking prevalence in this year.

Following the implementation of this legislation, smoking rates rapidly decreased after the public was made aware of fines for those smoking in smoke-free areas. However, the main significance of the legislation was that it required those implementing the legislation to carry out a related suite of policies in order to achieve expected effects. This point is supported by the continued decline in smoking prevalence shown in both our results from the 2005, 2009 and 2013 surveys. It was evident that high initiation rates in 2005 survey (18–24 years old) was reflected in prevalence in 25–39 years in the 2009 survey (possible cohort effect). However, the prevalence dropped significantly in the young adults in 2013.

A NTD 5 (~0.16USD) surcharge was added to each cigarette packet only from 2002 (and increased to NT$10 in 2006). Manpower and funds were made available for promotion of outpatient smoking cessation clinics in 2002, followed by a quit line (2003), smoke-free schools (2003), smoke-free workplaces (2003), and smoke-free restaurants (2004). We found that smoking prevalence did not change greatly between 2001 and 2005. However, there was a statistically significant change observed in smoking cessation rates over this time. We found that the rate of smoking cessation in males aged 18 years and over in 2005 was significantly higher than that in 2001, particularly in those aged 40–64 years. In addition, rates of smoking cessation significantly increased in 2009 in those aged 18 years and over in both sexes and across all age groups. This confirmed or first hypothesis on the increasing smoking cessation rate in older adults after the implementation of the 2007 tobacco control. Figure 3 showed the total sale of cigarettes decreased sharply in 2008, when the THPA officially passed. Figure 3 also showed the increased of cigarette price (from 35 NTD/pack in 2005 to 55NTD/pack in 2009). The higher surcharge placed on tobacco products as part of the government’s tobacco control work. Thus, reduced smoking rate and increased smoking cessation rate.

The quit ratio almost doubled in men aged 25–39 and 40 or older from 2005 to 2009. Even though there was a big improvement, the quit ratios were much lower than those in the US [24]. Using the same survey NHIS, the US reported about 80 % quit in those aged 65+, >50 % quit among those aged 45–64, ~40 % in those aged 25–44 between 2005 and 2012 [24]. It was possible the high rate of quitting between the surveys which has not been seen in countries that have had declining prevalence for many years – which might be associated with quitting among those who were not too dependent on cigarettes in the US. Nevertheless, it implied that we have a big room for improvement in smoking cessation. Previous researches [25–29] have found that factors associated with smoking cessation include marital status, education level, retirement status, socioeconomic status, age, level of nicotine addiction, health status, and family support. Similarly, in our logistic regression analysis we found that after controlling for other variables, the chance of smoking cessation actually increased with age, higher education level, and in higher family income. The price of tobacco products is another important factor. High tobacco prices make women, and men with lower incomes, quit smoking.

**Fig. 4:**
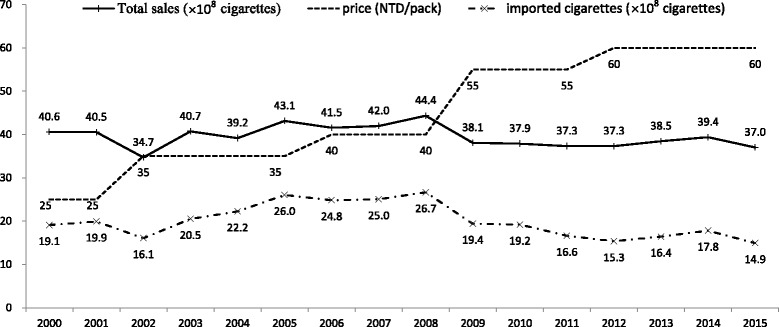
The total sales of cigarettes (including domestic and foreign cigarettes, ×10^8^), cigarette price (NTD/pack), and imported ones

The smoking rate was slightly different in females. It was much lower than the male rates. The low smoking rate in females has something to do with the culture. In the old Chinese culture, female smoking is not acceptable. More and more females started to smoke nowadays. Chinese female smokers might have something to do with showing off social economic status; and some young women might use smoke to control weight. The sharp increase of smoking in females in 2005 was corresponding to the time Taiwan joined the World Trade Organization (WTO). The imported cigarettes started to rise in 2002, and reached the peak at 2005 (from 16.1 to 26 × 10^8^ cigarettes, about 61.5 % increase) (Fig. 4). Even though the 1997 regulation restricted the advertisement of imported cigarettes to 120 times in one magazine per year for each brand of cigarette, the merchants used many other ways to promote cigarettes, such as sponsoring activities, including cigarettes in selling other goods like watches or coffee. It was until 2009, the THPA banned the advertisements in magazines and the inclusion of cigarettes in selling other goods. It was possible that females were attracted by the advertisements of imported cigarettes during that time. After the implementation of THPA, female smoking rate, total and imported sales of cigarettes decreased. This confirmed our second hypothesis that smoking rates decreased in young women after THPA ban the cigarettes’ advertisements.

We found that the greatest decrease in exposure to secondhand smoke occurred in the workplace. However, exposure to secondhand smoke in non-smoking men increased in friend’s and relative’s houses. In women, exposure to secondhand smoke decreased in the home but increased in friend’s and relative’s houses. After the ban of smoking in indoor public areas, smokers were likely to smoke in home. The exposure to secondhand smoke was increased in outdoor public areas. Since we did not have consistent data in this respect, we only reported the overall exposure and the areas were consistently asked. We only analyzed the exposure of non-smokers. Recall bias might occur among non-smokers who did not like smoke. In logistic regression analysis, after controlling for age, education level, marital status, individual income, household income, smoking, alcohol consumption, and betel nut chewing, we found that exposure to secondhand smoke in Taiwan only significantly decreased in women in 2009. It was significantly decreased in 2013 in men and women. This confirmed our third hypothesis about exposure to secondhand smoke among nonsmokers especially in workplace.

Experience in other countries demonstrates the importance of a multi-faceted approached. In Australia, although the tobacco tax underwent a large increase from 12.5 % to 35 % in 1983, smoking prevalence only declined when greater funds were put towards tobacco harm education [30]. Thailand’s experience in tobacco prevention also shows the importance of combining strategies [31]. In Thailand anti-smoking effects included: (1) increasing the tobacco tax; (2) expanding smoke-free areas; and (3) banning tobacco advertising, banning sales to those aged under 18 years, banning assistance for tobacconists, and the addition of graphic warning labels to cigarette packets. We cannot isolate the effect of TPHA from tobacco surcharge. Therefore, we believe that effective changes in smoking behaviour and reduction in smoking rates requires multifaceted and comprehensive smoking cessation services and strengthening of enforcement in addition to enacting strict tobacco hazard prevention legislation. This is in line with the recommendations of the Task Force on Community Preventive Services (TFCPS). They did a systematic review based on the recommendations by the TFCPS regarding the use of selected intervention on the aspects of effectiveness, applicability, other effects, economic evaluations, and barriers was carried out [32]. The task force have presented many achievements in all the aspects (http://www.thecommunityguide.org/tobacco/comprehensive.html).

There were several limitations in this study. All items were asked to individuals. It would subject to recall bias, especially those who quit smoking. The duration of cessation was not known. Gilpin et al. indicated that proxy might not know the smoking status of the respondents [33]. In our survey, proxy was only used in those who were: (1) severely ill or too frail to answer; (2) having cognitive problems; (3) having hearing or speaking problems; or (4) dementia. Among those aged 18–64 years old, 2 % of the respondents was proxy in year 2001. The rest of surveys, less than 1 % of the respondents were proxy. The use of proxy was high in those aged 65 or older, about 10 %. The discrepancies might appear in elderly. On the other hand, proxy data might have problem in questions relate to other information on smoking such as cigarettes per day, when quit attempts were made etc. Proxy reporting of these data could introduce significant bias. The conservative approach in this study is a strength. In 2005 and 2009, self-administered questionnaires were used as well as face-to-face interview. When comparing the report of smoking, the rate of smoking in males was 2–3 % lower in the face-to-face interview than the self-administered, whereas it was 0–3 % different in females (data not show). The other potential source of recall bias was the duration of smoking/quit. Cessation rate (Quit ratio) increased with time or age [16]. As the smoking duration or age increased, smokers had higher chance to quit. Also, the proportion of ever smokers in a birth cohort decreased with age. It was likely that death due to smoking increased and long term quitters, particularly those with short smoking histories, may have reclassified themselves as never smokers. The other limitation was that the cross-sectional nature of the data might not allow us to examine the changes of behavior over time. The strength of this study was that NHIS took national representative samples at a specific point of time and provide the best estimate of where population is at that time.

## Conclusion

This study utilized four waves of NHIS to examine the time trend of smoking, quitting, and exposure to secondhand smoking. Early anti-smoking legislation in Taiwan might have raised the awareness of the harm of smoking. It was until 2009 when manpower and resources were in place, smoking rate and exposure to secondhand smoke in workplace decreased, and quit ratio increased. The multi-faceted approach, the THPA, is the way to control cigarette smoking.

## Additional files

Additional file 1: Questions on smoking, secondhand smoking. (DOC 26 kb)
Additional file 2: SAS-callable SUDAAN program for this study. (DOC 56 kb)
