# Supplementary material for: A population study on the time trend of cigarette smoking, cessation, and exposure to secondhand smoking from 2001 to 2013 in Taiwan
Source: Popul Health Metr. 2016 Nov 5;14:38. doi: 10.1186/s12963-016-0109-x (PMC5097365; doi:10.1186/s12963-016-0109-x)
Supplement: Additional file 1: — Questions on smoking, secondhand smoking. (DOC 26 kb) [file 12963_2016_109_MOESM1_ESM.doc]

Supplement 1. Questions on smoking, secondhand smoking

D4. Have you ever smoked?

□1 Never 【Go to D5】

□2 Only a few times 【Go to D5】

□3 Yes, I have smoked less than five packs (100 cigarettes) from before ‘til now. 【Go to D4a】

□4 Yes, I have smoked more than five packs (100 cigarettes) from before ‘til now. 【Go to D4a】

D4a. When did you start smoking? Since I was ___ years old

D4b. How many years have you smoked? About___ years___
 months

□77 over 20 years

D4c. Have you smoked recently? (in past 30 days)

□1 I smoke (almost) every day, approximately ____cigarettes
 per day

□2 I often or sometimes smoke, approximately _____ cigarettes
 per month

□3 I have quit smoking
 How long have you quit smoking? Years Months.

Exposure to secondhand smoke (2005)

E5e. In the past week, have you ever exposed to secondhand smoke?

□0 No [Go to E5f]

□1 Yes

E5e_1. Where were you exposed to secondhand smoke? [multiple choices
 are allowed]

□a. Home or living places; □b. Relatives’ or friends’ places;

□c. School; □d. work place;

□e. Restaurant;

□f. Other public places.

E5e. In the past week, have you ever exposed to secondhand smoke? (2009, 2013)

□0 No [Go to E5f]

□1 Yes

E5e_1. Where were you exposed to secondhand smoke? [multiple choices
 are allowed]

□a. Home or living places; □b. Relatives’ or friends’ places;

□c. School; □d. work place;

□e. Other indoor public places;

□f. Other outdoor public places.
