# Supplementary material for: A population study on the time trend of cigarette smoking, cessation, and exposure to secondhand smoking from 2001 to 2013 in Taiwan
Source: Popul Health Metr. 2016 Nov 5;14:38. doi: 10.1186/s12963-016-0109-x (PMC5097365; doi:10.1186/s12963-016-0109-x)
Supplement: Additional file 2: — SAS-callable SUDAAN program for this study. (DOC 56 kb) [file 12963_2016_109_MOESM2_ESM.doc]

Supplement 2. SAS-callable SUDAAN program for this study.

**data** nhis2013.y4all ; set cc2;

if year=**2001** then survey=**1**;

if year=**2005** then survey=**2**;

if year=**2009** then survey=**3**;

if year=**2013** then survey=**4**;

if **11**<=age_1<**18** then age_5=**1**;

else if **18**<=age_1<**25** then age_5=**2**;

else if **25**<=age_1<**40**then age_5=**3**;

else if **40**<=age_1<**65** then age_5=**4**;

else if age_1>=**65** then age_5=**5**;

if **11**<=age_1<**18** then age_18=**1**;

else if **18**<=age_1<**130** then age_18=**2**;

if mar_1=**1** then mar_new=**1**;

if **2**<=mar_1<=**6** then mar_new=**2**;

if mar_1=**7** then mar_new=**3**;

if **0**=<edu<=**12** then edu_1=**1**;/*education level*/

else if edu in ( **0** **20** **21** **90**) then edu_1=**1**;

else if **13**=<edu<=**19** then edu_1=**2**;

else if **91**=<edu<=**92** then edu_1=**2**;

if homein=**0** then income_p=**1**;

if **0**<=homein<=**2** then income_p=**1**;/*individual monthly income*/

else if **3**<=homein<=**4** then income_p=**2**;

else if homein=**5** then income_p=**3**;

else if **6**<=homein<=**9** then income_p=**4**;

if home_in_m=**1** then income_h=**1**;/*family income*/

if **2**<=home_in_m<=**4** then income_h=**2**;/*family income*/

if **5**<=home_in_m<=**7** then income_h=**3**;

/*define smoking status, 1:none; 2: smoker; 3:quit 2*/;

if smok in ( **0** **1** **2** ) then smoker=**1**; else if smok in (**3**) and smok_now in (**1** **2**) then smoker=**2**;

else if smok in (**3**) and smok_now in (**3**) then smoker=**3**;

/* define secondhand smoking 1:none; 2: yes*/;

if year=**2005** and T_smok=**1** then home=**1**;

if year=**2005** and T_smok=**1** then oth_home=**1**;

if year=**2005** and T_smok=**1** then school=**1**;

if year=**2005** and T_smok=**1** then workplace=**1**;

if year=**2005** and T_smok=**1** then rest=**1**;

if year=**2005** and T_smok=**1** then pubic=**1**;

if year in (**2009** **2013**) and T_smok=**1** then home=**1** ;

if year in (**2009** **2013**) and T_smok=**1** then oth_home=**1** ;

if year in (**2009** **2013**) and T_smok=**1** then school=**1** ;

if year in (**2009** **2013**) and T_smok=**1** then workplace=**1** ;

if year in (**2009** **2013**) and T_smok=**1** then pubic_in=**1**;

if year in (**2009** **2013**) and T_smok=**1** then pubic_out=**1**;

if home=**2** or oth_home=**2** or school=**2** or workplace=**2** then t_4_smok=**2**;

if home in (**1**) and oth_home in (**1**) and school in (**1**) and workplace in (**1**) then t_4_smok=**1**;

if year=**2005** and rest in (**1**) and pubic in (**1**) then t_public_smok=**1**;

if year in (**2009** **2013**) and pubic_in in (**1**) and pubic_out in (**1**) then t_public_smok=**1**;

if rest =**2** then t_public_smok=**2**;

if pubic=**2** then t_public_smok=**2**;

if pubic_in=**2** then t_public_smok=**2**;

if pubic_out=**2** then t_public_smok=**2**;

if year=**2005** and t_public_smok in (**1** **2** ) then t_SHS=t_public_smok;

if year in (**2009** **2013**) and pubic_in in (**1** **2**) then t_SHS=pubic_in;

if smoker in (**1** **3**) then smoker_now=**1**;/*define smoking status, 1=no*/

if smoker=**2** then smoker_now=**2**;/*define smoking status, 2=yes*/

if smoker in (**2**) then smoker_quit=**1**;/*define quit, 1=no*/

if smoker=**3** then smoker_quit=**2**;/*define quit, 2=yes*/

if smoker=**1** then smoker_once=**1**;/*define ever-smoker, 1=never*/

if smoker in (**2** **3** ) then smoker_once=**2**;/*define ever-smoker, 2=ever*/

if sex=**3** then sex=**.**;

if **0**<=smok_no<=**60** then smok_no_1=smok_no;

if **60**<smok_no<=**500** then smok_no_1=**60**;

if smoker=**2** and **0**<=smok_no_1<=**5** then smok_no_level=**1**;

else if smoker=**2** and **5**<smok_no_1<=**10** then smok_no_level=**2**;

else if smoker=**2** and **10**<smok_no_1<=**20** then smok_no_level=**3**;

else if smoker=**2** and **20**<smok_no_1<=**80** then smok_no_level=**4**;

if now_UB<**0** then now_ub=**.**;

if now_ub=**1** then city1=**1**;

else if **2**<=now_ub<=**3** then city1=**2**;

else if **4**<=now_ub<=**7** then city1=**3**;

now_cc1=now_cc***1**;

if **1**=<now_cc1<=**7** then nowcc=now_cc1;

if now_cc1=**21** then nowcc=**8**;

if **23**=<now_cc1<=**37** then nowcc=now_cc1-**14**;

if smoker in ( **1** **2** ) then smoker_q_time=smoker;

else if smoker in (**3**) and smok_qy>=**1** then smoker_q_time=**4**;

else if smoker in (**3**) and smok_qtry=**2** then smoker_q_time=**4**;

else if smoker in (**3**) then smoker_q_time=**3**;

/*define quit time, 1: < 1 year, 2:1 year +*/;

if smoker_q_time in ( **2** **3** ) then smoker_q_year=**1**; if smoker_q_time in ( **4** ) then smoker_q_year=**2**;

if acl_last in (**2** **3** **4** **5**) then acl_y=**2**;

if acl_last in (**0** **1**) then acl_y=acl_last;

if acl_1 in (**2** **3** **4** **5**) then acl_m=**2**;

if acl_1 in (**0** **1**) then acl_m=acl_1;

if acl_1 in (**2** **3** **4** **5**) then acl_y=**2**;

if acl_1 in (**0** **1**) then acl_y=**1**;

run;

'demographic characteristics'

Sudaan:

data kk;set nhis2013.y4all;

if age_5>**1**;

age_4new=age_5-**1**;

if **1**<=survey<=**4**;

if istrata>**0**;

if psu_id>**0**;

if sex>**0**;

run;

**proc** **sort** data=kk;by survey icounty istrata psu_id ; **run**;

**proc** **crosstab** data=kk filetype=sas design=wor;

nest survey icounty istrata psu_id;

totcnt _zero_ _zero_ psu _minus1_;

subgroup sex survey age_4new city1 edu_1 income_h income_p mar_new work_now smoker drink betel_y ;

level **2** **4** **4** **3** **4** **3** **2** **3** **2** **3** **2** **2**;

tables sex*survey*(age_4new city1 edu_1 income_h mar_new work_now smoker drink betel_y);

weight wt_n;

test chisq ;

**run**;

Sudaan:'comparing categorical data in 2001、2005、 2009、2013';

**data** kk;set nhis2013.y4all;

if age_5>**1**;

age_4new=age_5-**1**;

if istrata>**0**;

if survey>**0**;

if psu_id>**0**;

if sex>**0**;

run;

**proc** **sort** data=kk;by survey icounty istrata psu_id ; **run**;

**proc** **crosstab** data=kk filetype=sas design=wor;

nest survey icounty istrata psu_id;

totcnt _zero_ _zero_ psu _minus1_;

subgroup survey sex smoker_now smoker_quit age_4new city1 edu_1 income_h;

level **4** **2** **2** **2** **4** **3** **2** **3**;

tables sex*age_4new*survey*(smoker_now smoker_quit);

weight wt_n;

test chisq ;

**run**;

Sudaan:'comparing secondhand smoking-2005、209 、2013 ';

**data** kk;set nhis2013.y4all;

if age_5>**1**;

age_4new=age_5-**1**;

if istrata>**0**;

if survey=**2** then survey1=**3**;

if survey=**3** then survey1=**1**;

if survey=**4** then survey1=**2**;

if psu_id>**0**;

if sex>**0**;

run;

**proc** **sort** data=kk;by survey icounty istrata psu_id ; **run**;

**proc** **crosstab** data=kk filetype=sas design=wor;

nest survey icounty istrata psu_id;

totcnt _zero_ _zero_ psu _minus1_;

subgroup smoker_now survey1 sex smoker_quit home oth_home school workplace t_public_smok t_smok;

level **2** **3** **2** **2** **2** **2** **2** **2** **2** **2**;

subpopn smoker_now=**1**;

tables sex*smoker_now*survey1*(home oth_home school workplace t_public_smok t_smok);

weight wt_n;

test chisq ;

**run**;

Sudaan:'Table 3. Factors associated with smoking in 2001 2005 2009 2013';

**data** kk;set nhis2013.y4all;

if age_5>**1**;

age_4new=age_5-**1**;

if istrata>**0**;

if psu_id>**0**;

if sex>**0**;

if smoker_now>**0** then smoker_now1=smoker_now-**1**;

run;

**proc** **sort** data=kk;by survey icounty istrata psu_id; **run**;

**proc** **rlogist** data=kk design=wor;

nest survey icounty istrata psu_id;

totcnt _zero_ _zero_ psu _minus1_;

subpopn sex=**2** ;

class age_4new survey edu_1 NOW_UB mar_new income_h income_p betel_y acl_y city1 work_now ;

weight wt_n;

reflevel age_4new=**2** edu_1=**1** survey=**3** income_h=**2** work_now=**1** city1=**1** mar_new=**1** betel_y=**1** acl_y=**1**;

model smoker_now1= age_4new edu_1 city1 mar_new income_h work_now betel_y acl_y survey;

**run**;

Sudaan:' factors associated with male quit smoking 2001 2005 2009 2013';

**data** kk;set nhis2013.y4all;

if age_5>**1**;

age_4new=age_5-**1**;

if istrata>**0**;

if psu_id>**0**;

if sex>**0**;

if smoker_quit>**0** then smoker_quit1=smoker_quit-**1**;

run;

**proc** **sort** data=kk;by survey icounty istrata psu_id; **run**;

**proc** **rlogist** data=kk design=wor;

nest survey icounty istrata psu_id;

totcnt _zero_ _zero_ psu _minus1_;

subpopn sex=**2** ;

class age_4new survey edu_1 NOW_UB mar_new income_h income_p betel_y acl_y city1 work_now ;

weight wt_n;

reflevel age_4new=**2** edu_1=**1** survey=**3** income_h=**2** work_now=**1** city1=**1** mar_new=**1** betel_y=**1** acl_y=**1**;

model smoker_quit1= age_4new edu_1 city1 mar_new income_h work_now betel_y acl_y survey;

**run**;

Sudaan:'Factors associated with males exposed to secondhand smoke in 2001 2005 2009 2013 ';

**data** kk;set nhis2013.y4all;

if age_5>**1**;

age_4new=age_5-**1**;

if istrata>**0**;

if survey=**2** then survey1=**1**;

if survey=**3** then survey1=**2**;

if survey=**4** then survey1=**3**;

if survey1>=**1**;

if psu_id>**0**;

if sex>**0**;

if t_smok>**0** then t_smok1=t_smok-**1**;

run;

**proc** **sort** data=kk;by survey icounty istrata psu_id; **run**;

**proc** **rlogist** data=kk design=wor;

title 'secondhand smoking among non-smokers';

nest survey icounty istrata psu_id;

totcnt _zero_ _zero_ psu _minus1_;

subpopn sex=**2** and smoker_now=**1**;

class age_4new survey1 edu_1 NOW_UB mar_new income_h income_p betel_y acl_y city1 work_now smoker_now;

weight wt_n;

reflevel age_4new=**2** edu_1=**1** survey1=**1** income_h=**2** work_now=**1** city1=**1** mar_new=**1** betel_y=**1** acl_y=**1** smoker_now=**1**;

model t_smok1= age_4new edu_1 city1 mar_new income_h work_now betel_y acl_y survey1;

**run**;
